# Increasing seated reaction forces with lower body negative pressure

**DOI:** 10.1038/s41526-025-00512-w

**Published:** 2025-08-14

**Authors:** Suhas Rao Velichala, Jonathan Kim, Alan R. Hargens

**Affiliations:** 1 Department of Orthopaedic Surgery, University of California, San Diego, San Diego, CA USA; 2 Western University of Health Sciences, Pomona, CA USA

**Keywords:** Translational research, Biophysics, Medical research, Developing world

## Abstract

This study evaluates reaction forces and cardiovascular responses during seated lower body negative pressure (LBNP). Ten healthy subjects were exposed to randomized LBNP levels (−10 to −40 mmHg) while seated in a sealed chamber. Gluteal, foot, and total reaction forces, along with heart rate and blood pressure, were measured at each level. Reaction forces increased significantly with rising LBNP (*P* < 0.05), exceeding baseline at 10 mmHg and doubling by 30 mmHg. Cardiovascular parameters remained stable, indicating no acute hemodynamic risk. Force generation was dependent on LBNP amplitude and waist cross-sectional area. These findings suggest that seated LBNP is a safe and effective method to simulate Earth-like seated posture in microgravity, offering a promising countermeasure to mitigate musculoskeletal deconditioning and support gravitational adaptation during long-duration spaceflight.

## Introduction

With expeditions to space becoming longer, injuries and detrimental adaptations to microgravity become a more important matter. Prolonged exposure to microgravity leads to significant musculoskeletal deconditioning, including muscle atrophy and bone density loss, which pose challenges for astronauts during and after space missions^1^. Previous studies document that astronauts experience a myriad of health complications during prolonged exposure to microgravity, including vision impairment (SANS), bone loss, muscular atrophy, neurological decrements, and cardiovascular deconditioning. Astronauts exhibit cardiovascular adaptations even after short-term exposures to microgravity^2,3^. Less dangerous, but equally pressing, are the injuries and dysfunctional responses that astronauts endure upon return to Earth’s gravity.

Due to the microgravity of spaceflight, astronauts are unable to assume the weight bearing and postures to which humans have adapted in normal daily activities on Earth. On Earth, most people spend 10–12 h per day in a seated posture while working, resting, visiting others, waiting, eating, and driving. Upon return to Earth, many astronauts complain of discomfort and pain when trying to sit again after several months without doing so^4^. Thus, an understanding of forces on the body while sitting is necessary to develop adequate countermeasures during spaceflight and prevent loss of adaptation to gravity on Earth. Such countermeasures would ideally reproduce conditions and activities similar to those common to daily living on Earth. Using LBNP to simulate conditions found on Earth may provide the loading and avoidance of headward fluid shifts to counteract the effects of microgravity on the body and thus, better preserve the health and well-being of astronauts in space.

Prior studies demonstrate that microgravity conditions increase headward fluid shifts and eliminate gravitationally induced load bearing. Existing countermeasures, such as resistance and aerobic exercises, have been implemented to mitigate these effects; however, they may not fully replicate the gravitational loading experienced on Earth, leading to incomplete prevention of musculoskeletal degradation^5^. Prior studies document benefits of LBNP on many physiologic systems including those for cardiovascular, musculoskeletal, and ocular health^6,7^.

The aim of this study is to quantify reaction forces and cardiovascular parameters associated with sitting within LBNP on Earth with a view to understand the mechanism of weightbearing and safety of brief periods of LBNP in seated subjects. Essentially, this study evaluates LBNP as a way of simulating the reaction forces associated with sitting. We hypothesize that the mechanism by which the reaction forces are generated depends on the amplitude of negative pressure and waist cross-sectional area. It is important to note that using LBNP in a seated position on Earth produces greater ground reaction forces (RFs) than in space due to the RFs already produced by Earth’s gravity alone. Baseline RF measurements on Earth include the participants’ normal body weights whereas this is not the case for measurements taken in Space. Though previous studies demonstrate that LBNP effectively generates load bearing, no such study is undertaken in a seated position to understand seated reaction forces and associated cardiovascular responses^2,8^. Boda et al. 2000 finds extensive similarities between supine LBNP exercise and normal upright exercise, but does not explore seated LBNP as an additional concept to reproduce the most common daily “exercise” on Earth^9^. Lathers and Charles explore the effects of extended LBNP in both standing and seated subjects as a countermeasure against orthostatic intolerance and the de-adaptation response of the cardiovascular system in microgravity^10^. Extending beyond their previous work, our objective of this study is to understand the ability and safety of seated LBNP to reproduce the most common daily activity associated with living on Earth. We hypothesized that brief periods of seated LBNP safely increase reaction forces associated with sitting on Earth and thus support this novel concept to provide an integrated countermeasure for prolonged spaceflight.

## Results

### Participants

Ten subjects participated in the study and no adverse events occurred. All but two subjects were able to complete the study reliably and without any issue. Two subjects were disqualified because they were too slim, and an adequate airtight seal was not possible. Thus, 8 subjects were included in the data analyses.

### Reaction force results

Reaction forces increased and differed significantly with LBNP level as performed randomly (Fig. 2). At 0 mm Hg, −10 mmHg, −20 mmHg, and −30 mm Hg of LBNP, the groups (total, gluteal, feet) were significantly different from each other (*P* < 0.05). At all levels of LBNP, total RF changed significantly (*P* < 0.05). The subjects mean total percent body weight for baseline, −10 mmHg, −20 mmHg, −30 mmHg, and −40 mmHg were 98.0%, 123.3%, 152.4%, 192.3%, and 222.9%, respectively. Because cross-sectional areas of the subject were uniform, the mechanism by which the reaction force is generated depends solely on the amplitude of negative pressure.

**Fig. 1:**
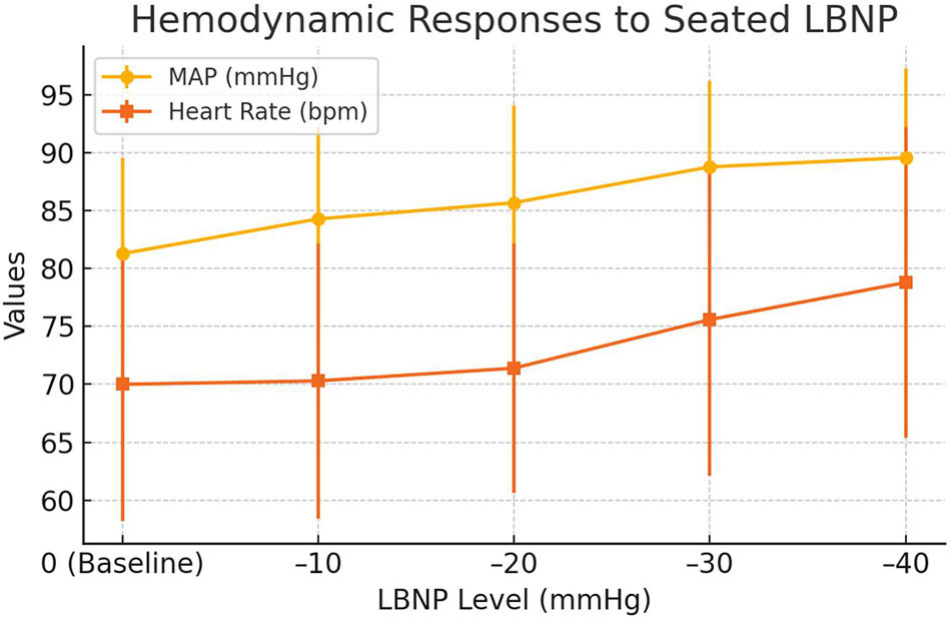
Hemodynamic responses progressively increase with rising levels of seated lower body negative pressure (LBNP). Both mean arterial pressure (MAP) and heart rate (HR) increased with increasing LBNP; error bars represent standard deviation. Statistical significance was observed starting at –20 mmHg for MAP and –10 mmHg for HR (*P* < 0.05).

### Hemodynamic results

For these brief exposures to LBNP, the subjects’ mean arterial pressures ± SD for baseline, −10 mmHg, −20 mmHg, −30 mmHg, and −40 mmHg were 81.3 ± 8.3 mmHg, 84.3 ± 7.9 mmHg, 85.7 ± 8.4 mmHg, 88.8 ± 7.4 mmHg, and 89.6 ± 7.7 mmHg, respectively. The subjects mean heart rates for baseline, −10 mmHg, −20 mmHg, −30 mmHg, and −40 mmHg were 70.0 ± 11.8 beats per minute (bpm), 70.3 ± 11.9 bpm, 71.4 ± 10.8 bpm, 75.6 ± 13.5 bpm, and 78.8 ± 13.4 bpm, respectively. Mean arterial pressures increased significantly (*P* < 0.05) starting at −20 mmHg. The heart rate increased significantly (*P* < 0.05) starting at −10 mmHg. Heart rate showed a statistically significant increase beginning at –10 mmHg (*P* < 0.05), while MAP rose significantly at –20 mmHg (*P* < 0.05) (Fig. 1) (Table 1). However, no values exceeded clinically relevant thresholds, supporting cardiovascular safety. Effect sizes (Cohen’s *d*) for heart rate and mean arterial pressure between baseline and –40 mmHg were 0.78 and 0.62 respectively, indicating moderate to large effects.

**Fig. 2:**
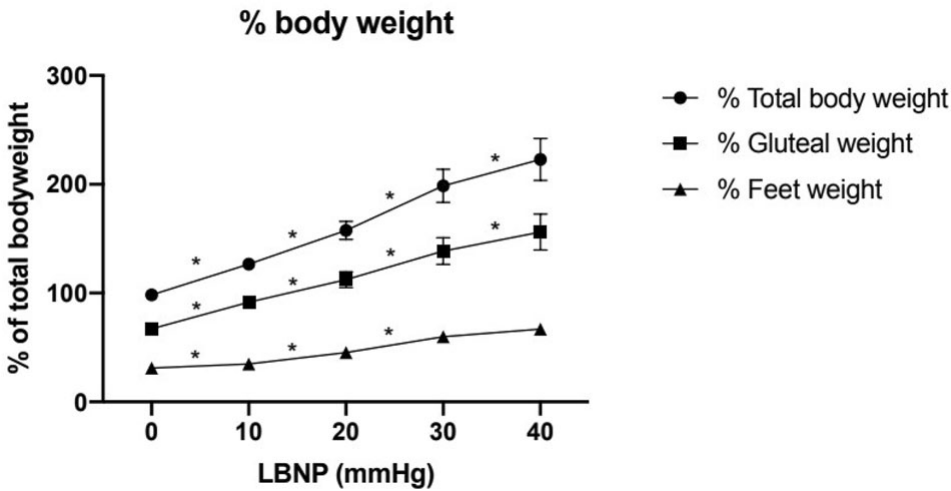
Ground reaction forces (RFs) increase significantly with rising levels of seated lower body negative pressure (LBNP). Differences between gluteal, foot, and total RFs were significant at 10, 20, and 30 mmHg; at 40 mmHg, only gluteal and total RFs differed from those at 30 mmHg.

**Table 1:** Hemodynamic responses at each level of seated LBNP

LBNP level (mmHg)	Mean arterial pressure (mmHg)	Heart rate (bpm)
0 (Baseline)	81.3 ± 8.3	70.0 ± 11.8
–10	84.3 ± 7.9	70.3 ± 11.9
–20	85.7 ± 8.4	71.4 ± 10.8
–30	88.8 ± 7.4	75.6 ± 13.5
–40	89.6 ± 7.7	78.8 ± 13.4

*Note: Heart rate increased significantly beginning at –10 mmHg (*P* < 0.05); mean arterial pressure increased significantly beginning at –20 mmHg (*P* < 0.05).

At 10 mmHg, 20 mmHg, and 30 mmHg of LBNP, the groups (gluteal, feet, total) are significantly different from each of the others. At 40 mmHg, only the gluteal and total forces were significantly different from the forces observed at 30 mmHg.

## Discussion

The primary findings of this study document for the first time that reaction forces (gluteal, foot, and total) increase significantly in response to moderate, increasing levels of LBNP. Furthermore, as hypothesized, the mechanism by which the reaction forces are generated depends on the amplitude of negative pressure and waist cross-sectional area as suggested by other studies of LBNP^2,11^. Moreover, because the increase in heart rates and blood pressures remained within physiologically safe ranges, these results support our hypothesis that LBNP safely and significantly increases reaction forces in seated subjects. Heart rates and blood pressures were measured in upright seated posture on Earth; and they would be higher than those obtained under actual microgravity conditions in which gravity is not present. This study is particularly novel as it introduces seated LBNP as a countermeasure that uniquely mimics the biomechanics of Earth’s most ubiquitous posture—sitting—while addressing the significant void in current countermeasure strategies for spaceflight. Unlike traditional aerobic and resistance exercises, seated LBNP directly targets and reproduces gravitational forces, which astronauts lose during extended missions. This direct simulation of load-bearing forces provides an entirely new avenue for mitigating musculoskeletal atrophy, headward fluid shifts, and post-flight readjustment challenges.

The increase in reaction forces in this first study of seated posture is consistent with previous studies that implemented LBNP to provide weightbearing in standing and supine postures^2,11^. The application of seated LBNP effectively simulates gravitational loading, thereby potentially mitigating musculoskeletal deconditioning by reproducing Earth-like mechanical stimuli in a microgravity environment^12^. LBNP is known to increase RFs in upright walking and running subjects, as well as increase load bearing in upright standing subjects^2,13^. Consistent with these prior studies, we observe that LBNP can be safely used in seated subjects to increase RFs. Gluteal forces, foot forces, and total forces all increased significantly with as little as 10 mmHg of LBNP. Importantly, we demonstrate for the first time that the mechanism by which the reaction forces are generated depends on the amplitude of negative pressure and waist cross-sectional area. The observed increase in ground reaction forces may have downstream effects on cardiovascular regulation. Increasing RFs likely promote venous return from the lower extremities, thereby modulating stroke volume and baroreceptor activity. These effects could contribute to the modest rise in MAP and HR observed in our study, consistent with previous reports on fluid redistribution during LBNP^14^. This connection warrants further exploration. Although heart rate and mean arterial pressure increased significantly at higher LBNP levels, the observed changes were modest and remained within physiologically safe ranges. On Earth, the upright seated posture used in this study differs from the microgravity environment of space, and the absence of significant cardiovascular changes may be attributed to gravitational effects inherent to the Earth’s surface. Importantly, this absence of significant changes underscores the safety of LBNP, even at higher pressure levels, making it a promising option for extended durations in space environments. Future studies should consider employing a supine seated posture to isolate the effects of LBNP from gravitational forces more effectively, potentially offering a more clear understanding of LBNP’s impact on fluid redistribution.

Low levels of LBNP are employed in many previous applications, both in moving and stationary subjects, and is generally considered safe and effective for counteracting headward fluid shifts and venous congestion in space^12^. Even so, there are certain risks and limitations, chiefly, the risk of syncope. However, this risk is seen in standing subjects, while supine subjects are typically at lower risk. Of the 10 subjects we evaluated for brief periods of seated LBNP at 10–40 mmHg, there were no presyncope symptoms or complaints of discomfort for any volunteer. However, the duration at which astronauts need to be exposed to LBNP as a countermeasure in space may present a syncopal problem and should be a potential follow-up study. Another limitation of the study is that we only employed an upright seated posture, which may not fully replicate the effects of LBNP experienced in a microgravity environment. We propose that future studies explore a supine-seated posture, as it may better simulate conditions of weightlessness while isolating the effects of LBNP, thereby providing more insight into potential cardiovascular adaptations. This study is a vital proof-of-concept, but the small sample size and lack of direct measurements of fluid shifts call for further exploration. Further research should explore the long-term effects of seated LBNP on musculoskeletal health during extended space missions to validate its efficacy as a sustainable countermeasure^13^. A larger cohort, combined with advanced imaging and biomarker analyses, would enhance the mechanistic understanding of how seated LBNP affects various physiologic systems. Long-term studies are also needed to evaluate its cumulative effects, particularly in a simulated microgravity environment. Lastly, optimizing the LBNP chamber design to accommodate varying anthropometric measurements would ensure broader applicability across diverse astronaut populations.

A key strength of this study is the randomization of LBNP levels; however, it lacks a detailed mechanistic understanding of the fluid shifts mentioned in the introduction. Including direct measurements or estimates of fluid shifts during seated LBNP would significantly strengthen our conclusions. We acknowledge that future studies should incorporate such measurements to provide a more comprehensive understanding of LBNP’s role in managing headward fluid shifts and related complications, such as venous thrombosis and SANS. One further limitation is that subjects of different heights and weights were studied in a single size chamber. Although seat height is adjusted accordingly, taller subjects may experience more difficulty with comfortable experimentation and cardiovascular testing.

Another limitation of this study is the restricted scope of hemodynamic monitoring. While heart rate and mean arterial pressure were recorded, other critical parameters such as stroke volume, cardiac output, and total peripheral resistance were not measured. These variables are essential for providing a comprehensive understanding of cardiovascular responses to LBNP, especially when evaluating its safety and efficacy as a countermeasure to microgravity-induced deconditioning. The use of non-invasive cardiac output monitoring technologies in future studies would allow for more detailed physiological profiling and mechanistic insight into how LBNP modulates cardiovascular dynamics across different pressure levels.

Ultimately, this study explores LBNP in the seated position for the first time as a possible integrated countermeasure for prolonged space flight. Our results document that all reaction forces (gluteal, feet, and total body) increase significantly with LBNP without significant changes in heart rate and blood pressure. As hypothesized, the mechanism by which the reaction forces are generated depends on the amplitude of negative pressure and waist cross-sectional area. The ability to simulate load bearing by seated LBNP may give insight into potential countermeasure therapies and devices that can be used to simulate sitting in microgravity. Implementing seated LBNP as a countermeasure could address the discomfort and musculoskeletal issues astronauts face upon return to Earth’s gravity, by maintaining the neuromuscular functions associated with sitting^4^. Adequate simulation of load bearing in the seated position in space may avoid the pain and inability to tolerate sitting commonly experienced by astronauts upon return to Earth.

Our results are consistent with previous studies that demonstrated LBNP’s ability to provide load bearing and Earth-like cardiovascular stress in upright posture. It is possible that seated LBNP in space adequately simulates the most common daily activity on Earth to counteract the unloading and deconditioning during prolonged space flight. Moreover, the recent findings of venous stagnation and thrombosis studies of headward fluid shifts during actual spaceflight suggest that LBNP is a viable countermeasure strategy for vision impairment related to headward fluid shifts^12^. As a pioneering exploration into seated LBNP, this study opens the door to a transformative approach in space medicine. Beyond its immediate implications for astronaut health, the findings could inspire novel interventions for managing conditions linked to prolonged immobility, such as deep vein thrombosis and vision impairment on Earth. Ultimately, the work provides a critical foundation for bridging the gap between Earth’s gravity-dependent activities and the unique challenges of microgravity, making it a key advancement in both aerospace and rehabilitation sciences.

## Methods

### Subjects

We recruited 10 healthy subjects, 6 males and 4 females, between the ages of 18–45 years to participate in the study. Subjects were informed on the background, protocol, and risks involved with participating in the study. Informed, written consent was obtained from all subjects. Prior to recruitment, approval was granted by the Institutional Review Board of the University of California, San Diego.

### Experimental protocol

The study was conducted in a quiet environment at room temperature. Subjects wore comfortable clothing to allow them to sit in their natural posture in the chamber. Once acclimated, baseline measurements were taken. Subjects refrained from caffeine and alcohol consumption for 24 h prior to the study. Lower Body Negative Pressures were applied in a randomized order and subjects were advised not to talk and were coached to relax during data collection. Each level of LBNP was applied for 2 min, after which ground reaction force and cardiovascular measurements were immediately recorded. The total experiment duration, including baseline and rest periods, was approximately 15–20 min per subject.

### Instrumentation and measurements

**Fig. 3:**
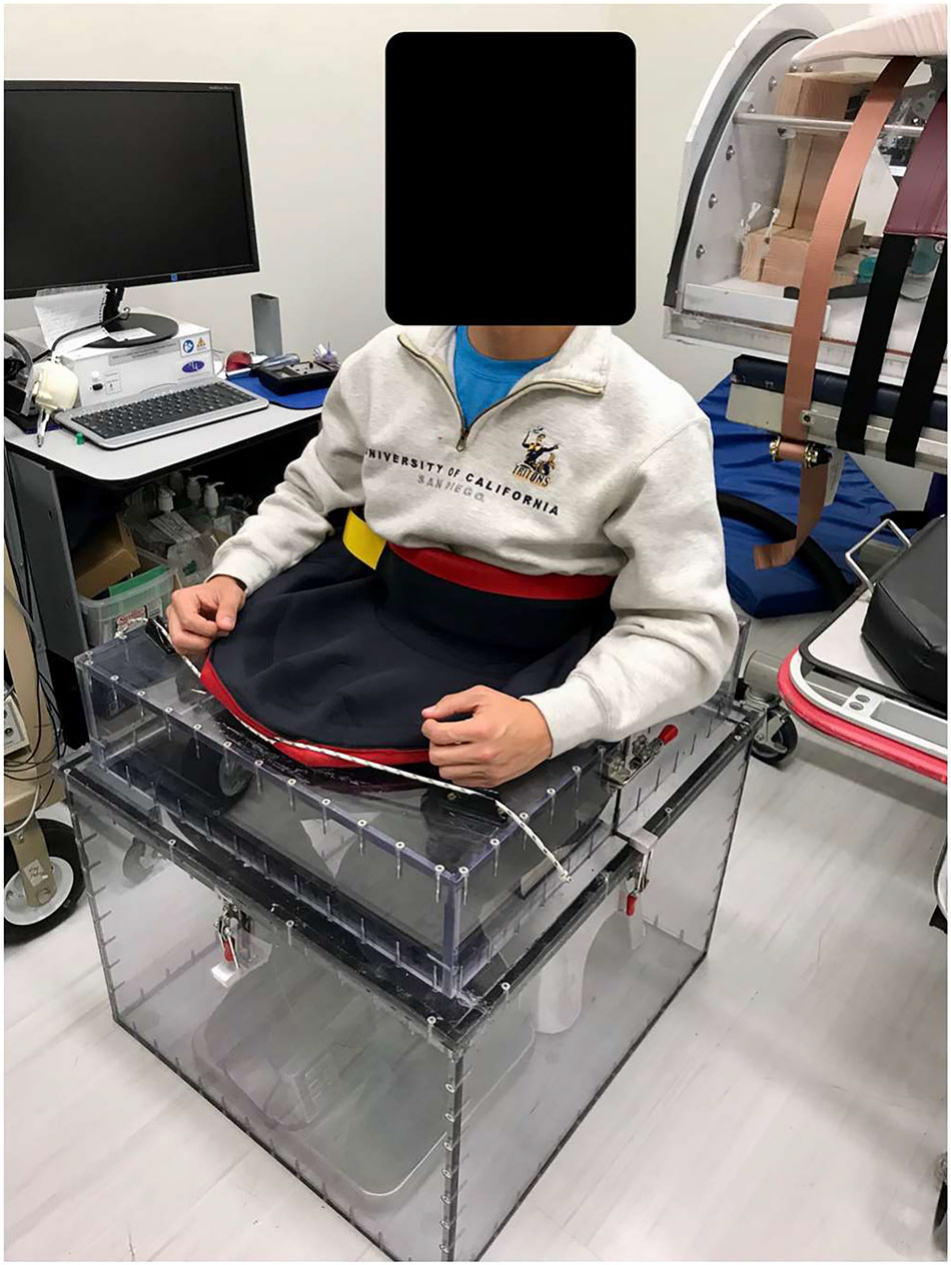
Seated LBNP chamber setup enables simultaneous measurement of ground reaction forces and cardiovascular responses. The participant is sealed at the iliac crest using a neoprene waist seal, with a scale placed under the feet and Tekscan sensors positioned under the seat to capture reaction forces.

*LBNP Chamber*. A chamber was constructed to contain a seated subject, sealed at the waist. Tek scan sensors (Pressure Mapping Sensor 9801) and a weight scale were positioned under the subject. The chamber was connected to a standard shop vacuum and pressure monitor. Subjects were seated with both feet on a weighing scale (read out of 0.1 kg) with their arms at their sides (Fig. 2). An airtight waist seal consisted of a neoprene skirt with an adjustable drawstring (Fig. 3). Once the volunteer was seated in a secure position, the vacuum applied varying levels of LBNP in random order (10 mm Hg, 20 mm Hg, 30 mm Hg, and 40 mm Hg).

### Cardiovascular data

Heart rate and blood pressure were measured using a standard blood pressure monitor at the end of each 2-min seated-LBNP interval.

### Force data

Tekscan sensors were used to track ground reaction forces under the seat. The sensors were calibrated before each trial. Accuracy and reproducibility were confirmed by ensuring that gluteal and foot forces added up to the subject’s total body weight.

### Sitting

All subjects began the experiment in sitting posture. The height of the seat was adjusted to ensure that all subjects sat with their knees and ankles at a 90-degree angle. Feet rested flat, directly on a scale. Subjects sat directly on top of TekScan pressure mapping sensors. Baseline total RF was calculated as the sum of the gluteal forces and foot forces. Subjects spent 3 min in the chamber at rest in order to obtain a baseline, control values. Each LBNP level was maintained for 2 min, allowing the subject to physiologically stabilize before cardiovascular and reaction force measurements were recorded. The entire protocol, including rest periods and transitions, lasted approximately 15–20 min per participant.

This study was considered a proof-of-concept investigation. No formal power analysis was conducted to calculate sample size, as the main objective was to assess feasibility and safety. Future studies should perform a power analysis to ensure adequate sample size for detecting subtle changes in cardiovascular and fluid-shift parameters. Future studies should include a power analysis to determine adequate sample sizes for detecting subtle changes in cardiovascular and fluid-shift parameters.

### Data analyses

Total ground reaction force is calculated as the sum of the Tek Scan component under the participants’ seat, as well as the scale component under the feet. These two readings are direct representations of the increase in RFs observed with increasing seated LBNP. Heart rate and blood pressure were measured using a standard blood pressure cuff. RF and cardiovascular data are compared between baseline (0 mmHg LBNP) and each interval of applied LBNP (10 mmHg, 20 mmHg, 30 mmHg, and 40 mmHg). Measurements at each level of LBNP were recorded after 2 min to allow participants to acclimate to a given LBNP level. As hypothesized, the mechanism by which the reaction forces were generated was evaluated by measurements negative pressure and waist cross-sectional area.

**Statistics** Means ± SD for each level of LBNP were compared using repeated-measures ANOVA with Greenhouse-Geisser correction to account for potential violations of sphericity. Post-hoc comparisons were performed using Bonferroni-adjusted *p*-values. Repeated measures ANOVA is robust to moderate deviations from normality, particularly in small samples, and is an appropriate method in this context^15^. Statistical significance was set at P < 0.05. Post-hoc analyses clarified comparisons between baseline and each LBNP condition, as well as between successive conditions.

Means ± SD for each level of LBNP were compared with the baseline control (0 mmHg) using repeated-measures ANOVA with a Greenhouse-Geisser correction to account for any sphericity violations. Post-hoc analysis was conducted using Bonferroni adjustments for multiple comparisons. Statistical significance was set at *P* < 0.05. All comparisons were made between each LBNP condition and the baseline, as well as between each successive condition (e.g., −10 mmHg compared to −20 mmHg).

## Data Availability

No datasets were generated or analysed during the current study.

## Code availability

The datasets generated and/or analyzed during the current study are available from the corresponding author on reasonable request. No custom code was used for data analysis in this study.
