# Author Correction: Increasing seated reaction forces with lower body negative pressure

**DOI:** 10.1038/s41526-025-00527-3

**Published:** 2025-09-13

**Authors:** Suhas Rao Velichala, Jonathan Kim, Alan R. Hargens

**Affiliations:** 1 Department of Orthopaedic Surgery, University of California, San Diego, San Diego, CA USA; 2 Western University of Health Sciences, Pomona, CA USA

**Keywords:** Translational research, Biophysics, Medical research, Developing world

Correction to: *npj Microgravity*10.1038/s41526-025-00512-w, published online 14 August 2025

In the sentence beginning ‘This study evaluates ground reaction forces...’ in this article, the text ‘evaluates ground reaction’ should have read ‘evaluates reaction’ and the abbreviation “GRF” should have read “RF”. The original article has been updated.

